# circSLCO1B7 suppresses the malignant progression of hepatocellular carcinoma via the miR-556-3p/DAB2IP axis

**DOI:** 10.18632/aging.205244

**Published:** 2023-11-24

**Authors:** Linling Ju, Qian Zhou, Qianyi Qi, Yongjun She, Weihua Cai, Yali Cao, Rujian Lu, Jianguo Shao, Lin Chen

**Affiliations:** 1 Medical School of Nantong University, Nantong University, Affiliated Nantong Hospital 3 of Nantong University, Nantong Third People`s Hospital, Nantong 226000, Jiangsu, China; 2 Department of Gastroenterology, Changshu Second People’s Hospital, Changshu 215500, Jiangsu, China; 3 Research Center of Clinical Medicine, Affiliated Hospital of Nantong University, Medical School of Nantong University, Nantong 226001, Jiangsu, China

**Keywords:** circSLCO1B7, hepatocellular carcinoma, miR-556-3p, DAB2IP, EMT

## Abstract

Circular RNAs (circRNAs) are noncoding RNAs with a circular colsed structure that play an important role in the occurrence and development of cancers. The functional mechanism of circRNAs as ceRNAs in hepatocellular carcinoma (HCC) and its effect on the invasion and metastasis of HCC need to be further studied. Five pairs of HCC tissues were selected for high-throughput sequencing, and 19 circRNAs with differential expression were obtained. The expression of circSLCO1B7 was obviously downregulated in 50 pairs of tumor tissues and plasma of HCC patients, which was closely related to the TNM stage, lymph node metastasis and tumor size. Cell functional experiments showed that circSLCO1B7 could inhibit cell growth, migration, invasion and promote cell apoptosis. In the regulatory mechanism, circSLCO1B7 sponged miR-556-3p to regulate the expression of the downstream target gene DAB2IP and induced the Epithelial-mesenchymal transition (EMT) progression. Our results indicated that circSLCO1B7 significantly inhibits the metastasis of HCC via the miR-556-3p/DAB2IP axis. Thus, circSLCO1B7 is a good candidate as a therapeutic target.

## INTRODUCTION

Hepatocellular carcinoma (HCC) is one of common cancers in the digestive system. The morbidity of HCC is increasing year by year, and its mortality ranks fourth among cancer-related deaths. Most patients are diagnosed with HCC when they are in the middle or advanced-stage cancer, which may also be accompanied by metastasis [1, 2]. Early diagnosis and treatment are the key to improving the quality of life of HCC patients. Unfortunately, HCC is often definitely diagnosed in the middle and late stages. Currently, alpha-fetoprotein (AFP) is the main serological indicator for the diagnosis of HCC, but its diagnostic accuracy is only approximately 60% [3, 4]. The occurrence and development of HCC is a multi-step, multi-stage process, and there are changes in different signaling pathways and gene expressions at each stage [5]. Therefore, it is of great significance to find new diagnostic markers for HCC and explore its invasion and metastasis mechanism to prolong the life of patients.

CircRNAs (circular RNAs), as oncogenes or tumor suppressor genes, can effectively regulate many biological behaviors, such as cell apoptosis, proliferation, invasion and migration [6–8]. The closed loop structure of circRNAs is formed by reverse splicing of pre-mRNA. Unlike linear RNAs, circRNAs without 5′ caps and 3′ polyadenylated tails are more stable, which keep themselves resistant to RNase R enzymes [9]. With the development of biological technology, many studies have shown that circRNAs exert many important functions, such as miRNA sponges, protein-binding regulation, gene transcriptional regulation and translation [10–12].

On one hand, circRNAs as oncogenes can promote the progression of cancers. For example, circRNA Cdr1as was highly expressed in HCC tissues compared to normal tissues. CircRNA Cdr1as turned normal liver cells to hepatocellular carcinoma cells and could sponge miR-1270 to regulate AFP level [13]. CircFOXK2 was highly expressed in advanced-stage breast cancer tissues. The higher the expression of circFOXK2 is, the higher the chance of lymph node metastasis and invasive histological type are. CircFOXK2 was positively related to metastasis of breast cancer [14]. On the other hand, circRNAs as tumor suppressor genes can inhibit the progression of cancers. CircNDUFB2 was downregulated in non-small cell lung cancer (NSCLC) tissues, and negatively correlated with tumor size and lymph node metastasis. CircNDUFB2 facilitated degradation of insulin-like growth factor 2 mRNA-binding proteins and played a key role in anti-tumor immunity [15]. CircDIDO1 was downregulated in gastric cancer tissues and its low level was associated with a larger tumor size and distal metastasis. CircDIDO1 inhibited the activity of poly ADP-ribose polymerase 1 through encoding protein [16].

In recent years, more and more studies have been conducted on the role of circRNAs in HCC. For example, circSLC3A2, as an oncogene gene, promoted the progression of hepatocellular carcinoma to sponge miR-490-3p and regulated the expression of protein phosphatase Mg^2+^/Mn^2+^ dependent 1F [17]. Hsa_circ_0008367 could interact with ALKBH5 to regulate the mechanism of iron death induced by sorafenib and could also affect autophagy and ferritin autophagy [18]. Sheng et al. found that circUBAP2 was highly expressed in HCC tissues and could promote the metastasis and invasion of HCC cells. circUBAP2 could sponge miR-1294 to regulate the expression of c-Myc. CircUBAP2 may be a potential biomarker for evaluation of HCC prognosis [19]. High expression of circMET was closely related to survival of HCC patients. CircMET could promote epithelial-mesenchymal transition, enhance immunosuppression and regulate tumor microenvironment. CircMET promoted the malignant progression of HCC via miR-30-5p/Snail/ dipeptidyl peptidase 4/CXCL10 axis [20].

This study was intended to explore the expressions of circSLCO1B7 in the tissues and plasma of HCC patients and analyze the relationship between the expression of circSLCO1B7 and the clinical data of patients, such as age, sex, HBV infection, TNM stage, lymph node metastasis, tumor size and AFP. In terms of mechanism, it is necessary to explore the mechanism of circSLCO1B7 as a ceRNA in competitively binding with miRNA to regulate the expression of downstream target genes and clarify its effect on the malignant progression of HCC.

## RESULTS

### Expression and characteristics of circSLCO1B7

**Figure 1:**
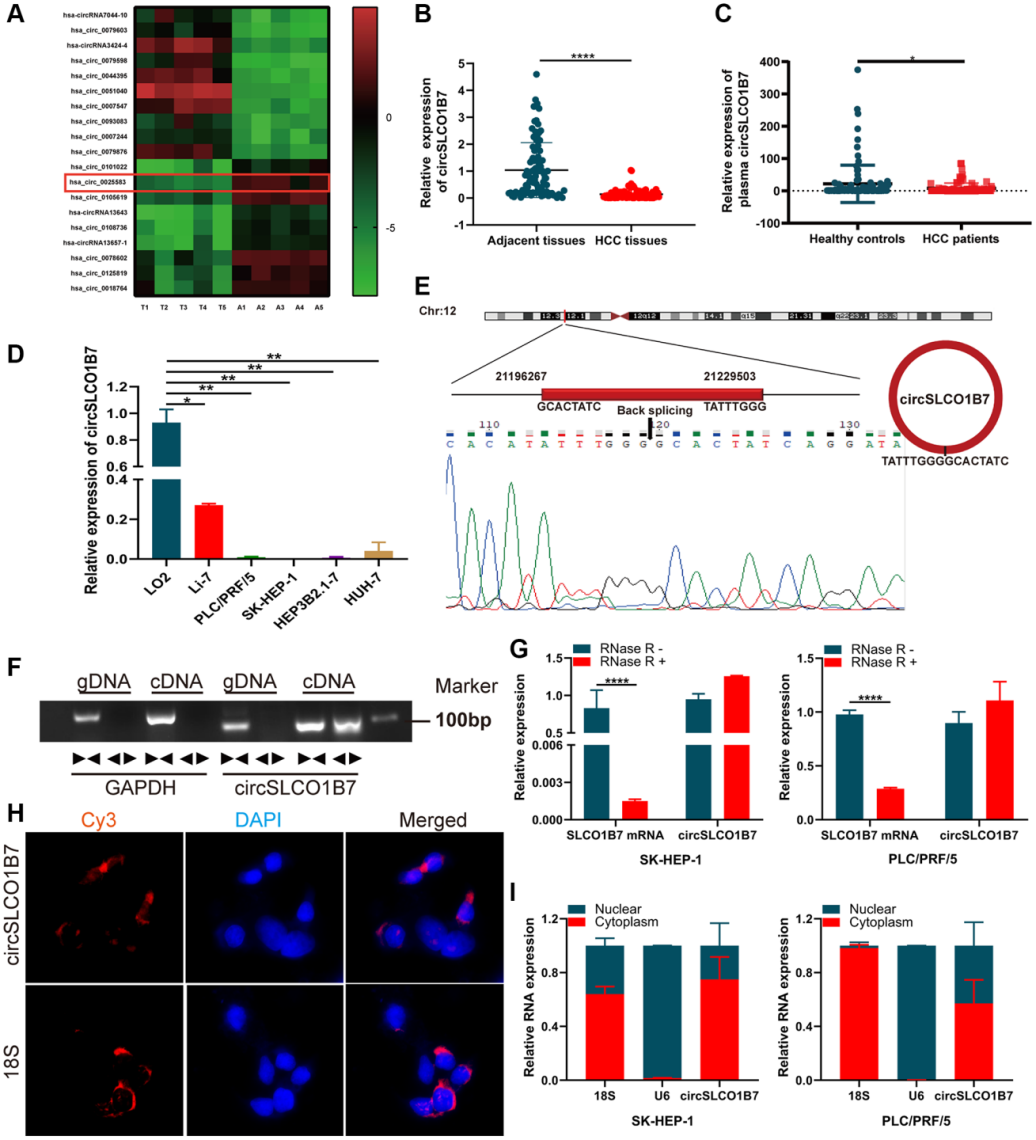
**The expression, structure and characteristics of circSLCO1B7 in HCC.** (**A**) The heatmap was used to screen circSLCO1B7 with low expression in HCC. (**B**) qRT–PCR analysis of the expression of circSLCO1B7 in HCC tissues. (**C**) qRT–PCR was used to detect the expression of circSLCO1B7 in the plasma of HCC patients. (**D**) The expression of circSLCO1B7 in HCC cells (SK-HEP-1, Li-7, PLC/PRF/5, Hep3B2.1-7 and HuH-7) compared to LO2 cells was detected by qRT–PCR. (**E**) Sequencing of qRT–PCR amplification products confirmed the circular site of circSLCO1B7. (**F**) Agarose gel electrophoresis showing the expression of circSLCO1B7 and GAPDH were amplified by divergent primers and convergent primers using cDNA and gDNA in SK-HEP-1 and PLC/PRF/5 cells. (**G**) The levels of circSLCO1B7 and SLCO1B7 in SK-HEP-1 and PLC/PRF/5 cells treated with RNase R were analysed by qRT–PCR. (**H**) FISH assay verified the location of circSLCO1B7 in PLC/PRF/5 cells. (**I**) qRT–PCR showed circSLCO1B7 was located in nuclear or cytoplasm. All data are presented as the means ± SD. ^*^P < 0.05, ^**^P < 0.01, ^****^P < 0.0001.

To explore the expression of circRNAs in HCC, we selectively used five pairs of adjacent normal tissues and cancer tissues for high-throughput sequencing. The results showed that there were more than 10,000 differentially expressed circRNAs. Among them, 19 circRNAs with obviously different expressions were listed, including 10 upregulated circRNAs and 9 downregulated circRNAs (Figure 1A). Has_circ_0025583 (termed circSLCO1B7) was predicted to be the most downregulated circRNA according to the sequencing results. As the sample size was further expanded, circSLCO1B7 was obviously downregulated in 50 pairs of adjacent normal tissues and HCC tissues (Figure 1B). Then, all 50 HCC patients were divided into two groups including the low circSLCO1B7 expression group (*n* = 25) and the high circSLCO1B7 expression group (*n* = 25) according to the median value of qRT–PCR analysis. The results showed that the differential expression of circSLCO1B7 was closely related to TNM stage, lymph node metastasis and tumor size. However, circSLCO1B7 was not related to patient age, sex, HBV infection and serum AFP (Table 1). To verify the diagnostic value for HCC, we collected plasma samples from 50 HCC patients and 50 normal healthy people. qRT–PCR confirmed that circSLCO1B7 was expressed at low levels in the plasma of HCC patients compared to that in normal healthy people (Figure 1C).

**Table 1:** The clinicopathological parameters of HCC patients.

**Characteristics**	**Cases**	**Expression**		***P* value**
		**Low**	**High**	
All cases	50	25	25	
Age, years				
<60	23	10	13	0.395
≥60	27	15	12	
Gender				
Male	36	17	19	0.529
Female	14	8	6	
HBV infection				
Yes	32	14	18	0.239
No	18	11	7	
TNM stage				
I	6	3	3	0.019
II	20	15	5	
III	10	4	6	
IV	14	3	11	
Lymph node metastasis				
Positive	16	12	4	0.039
Negative	34	13	21	
Tumor size, cm				
<5	36	15	21	0.017
≥5	14	10	4	
AFP, ng/ml				
<8.78	22	8	14	0.087
≥8.78	28	17	11	

Similar to the HCC tissues, the expression of circSLCO1B7 was significantly lower in HCC cells (Li-7, PLC/PRF/5, SK-HEP-1, Hep3B1.7 and HuH-7) than in normal liver cells (LO2) (Figure 1D). According to the data from circBase (http://www.circbase.org/), circSLCO1B7 was a fragment of the parent gene SLCO1B7 and was located on chr12:21196267-21229503, which had undergone reverse splicing to form a closed ring structure with a length of 1138 nt. The qRT–PCR product detected in the tissues was sequenced, and the head-to-tail splicing sequence of circSLCO1B7 was consistent with the expected splicing site (Figure 1E). On this basis, to verify the ring structure of circSLCO1B7, we constructed convergent and divergent primers according to the sequence. Since cDNA and genomic DNA (gDNA) could both exhibit head-to-tail phenomena, gel electrophoresis of the amplified product showed that circSLCO1B7 could be expressed in cDNA but not in gDNA (Figure 1F). To verify the stability of circSLCO1B7, qRT–PCR results showed that the expression of the linear parent gene SLCO1B7 was significantly inhibited in HCC cells treated by RNase R enzyme, while the expression of circSLCO1B7 remained basically unchanged. It was confirmed that circRNAs were not degraded by the RNase R enzyme (Figure 1G). With 18S as the control group, FISH assay (Figure 1H) and nuclear and cytoplasmic assay (Figure 1I) showed that most of circSLCO1B7 were located in cytoplasm.

### Effect of overexpression of circSLCO1B7 on proliferation, invasion, migration, apoptosis and cell cycle

**Figure 2:**
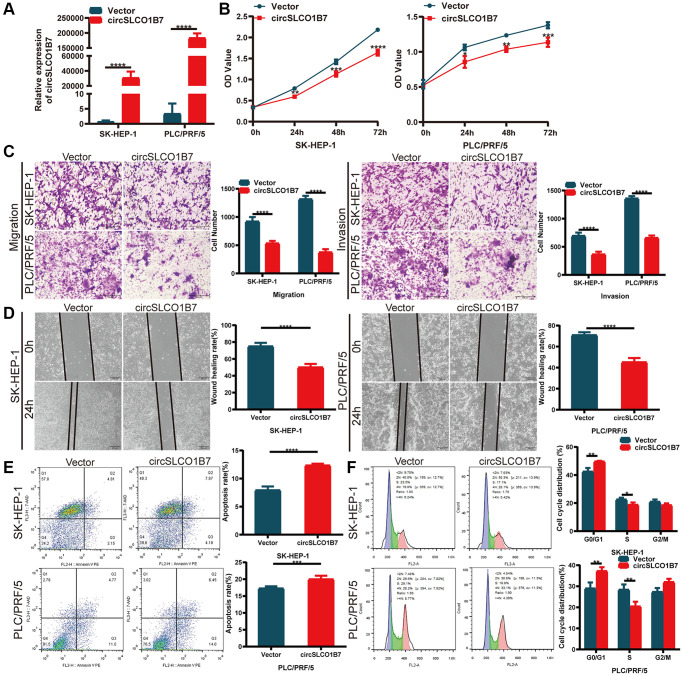
**Overexpression of circSLCO1B7 inhibited the progression of HCC cells.** (**A**) qRT–PCR analysis of the expression of circSLCO1B7 in HCC cells. (**B**) The CCK-8 assay was used to detect the proliferation ability of transfected SK-HEP-1 and PLC/PRF/5 cells. (**C**) The migration and invasion abilities of transfected SK-HEP-1 and PLC/PRF/5 cells were measured by Transwell assay. (**D**) A wound healing assay was performed to detect the migration levels of transfected SK-HEP-1 and PLC/PRF/5 cells. (**E**, **F**) After overexpression in SK-HEP-1 and PLC/PRF/5 cells, the apoptosis rate (**E**) and distribution of the cell cycle (**F**) were analysed by flow cytometry and compared to the vector group. All data are presented as the means ± SD. ^*^P < 0.05, ^**^P < 0.01, ^***^P < 0.001, ^****^P < 0.0001.

To explore the functions of circSLCO1B7 in HCC cells, we constructed a circSLCO1B7-overexpressing plasmid and a control vector, and then transfected them into SK-HEP-1 and PLC/PRF/5 cells. We verified that the overexpression efficiency of circSLCO1B7 was significant (Figure 2A). CCK-8 assays demonstrated that overexpression of circSLCO1B7 inhibited the proliferation of SK-HEP-1 and PLC/PRF/5 cells (Figure 2B). Transwell assays and wound healing assays were used to detect invasion and migration of HCC cells. The results revealed that overexpression of circSLCO1B7 significantly suppressed the invasion and migration of SK-HEP-1 and PLC/PRF/5 cells (Figure 2C, 2D). Flow cytometry was used to measure cell apoptosis and cell cycle distribution. The results showed that compared with the control group, overexpression of circSLCO1B7 significantly increased the apoptosis rate and arrested the cell cycle in the S phase (Figure 2E, 2F).

### Effect of knockdown of circSLCO1B7 on proliferation, invasion, migration, apoptosis and cell cycle

**Figure 3:**
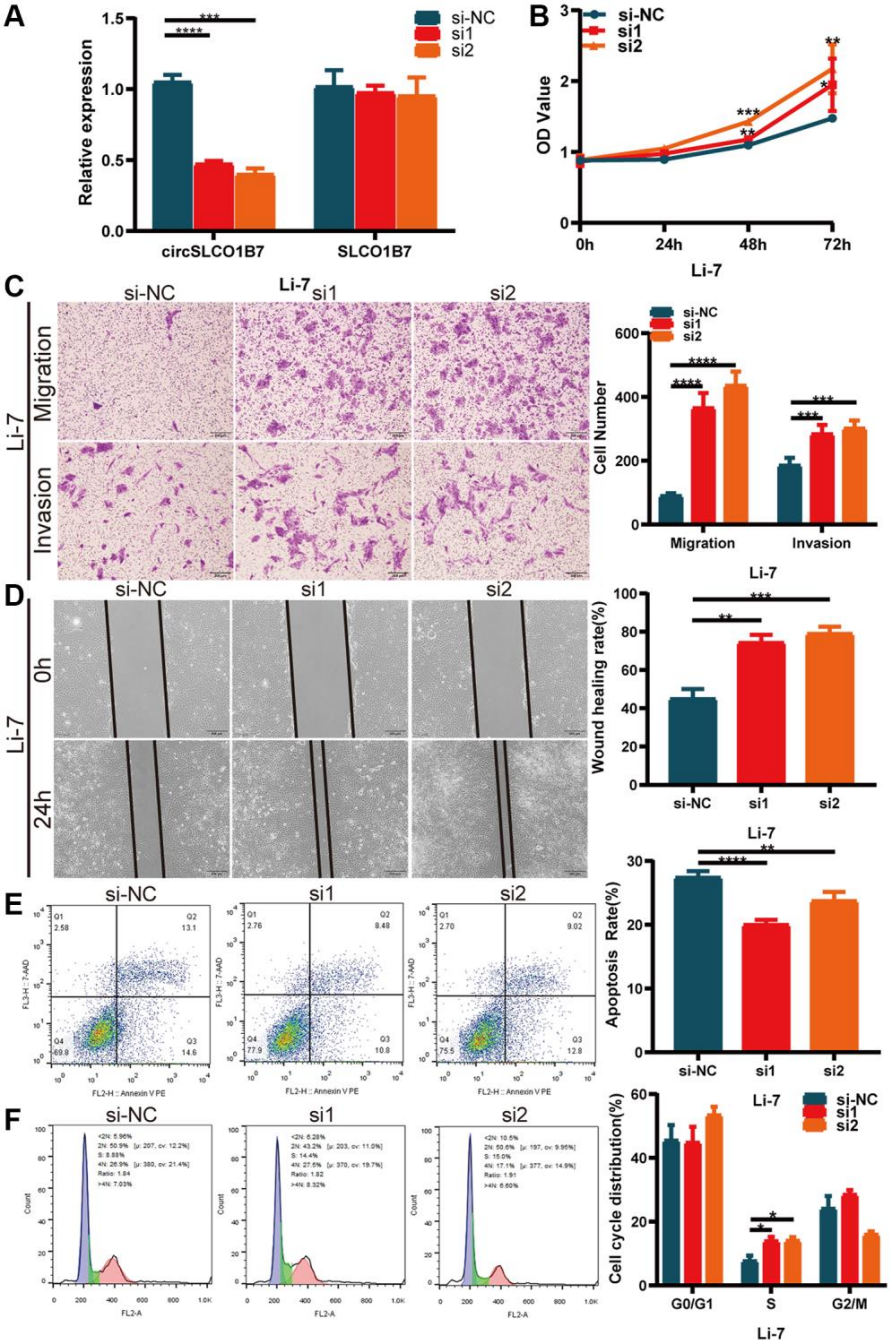
**Knockdown of circSLCO1B7 promoted the progression of HCC cells.** (**A**) qRT–PCR measured the knockdown efficiency of circSLCO1B7 in Li-7 cells transfected with si-NC, si1 and si2. (**B**) The CCK-8 assay was used to detect the proliferation ability of transfected Li-7 cells. (**C**) The migration and invasion ability of transfected Li-7 cells were measured by Transwell assay. (**D**) A wound healing assay was performed to detect the migration levels of transfected Li-7 cells. (**E**, **F**) The apoptosis rate (**E**) and distribution of the cell cycle (**F**) in transfected Li-7 cells were analysed by flow cytometry. All data are presented as the means ± SD. ^*^P < 0.05, ^**^P < 0.01, ^***^P < 0.001, ^****^P < 0.0001.

Next, we determined whether knockdown of circSLCO1B7 had the opposite biological function, and we designed two siRNAs (si1 and si2). Based on its expression in HCC cells, we transfected siRNAs into Li7 cells. qRT–PCR showed that the knockdown efficiency was satisfactory (Figure 3A). CCK-8 assays also showed that the proliferation of HCC cells increased after transfection with siRNAs (Figure 3B). Similarly, after knockdown of circSLCO1B7, the migration and invasion abilities of Li7 cells was enhanced compared with that of the normal control group according to the results of Transwell assays (Figure 3C) and wound healing assays (Figure 3D). Knockdown of circSLCO1B7 reduced the cell apoptosis rate and arrested the cell cycle in the G0/G1 phase, as determined by flow cytometry (Figure 3E, 3F).

### circSLCO1B7 functions as a miR-556-3p sponge

**Figure 4:**
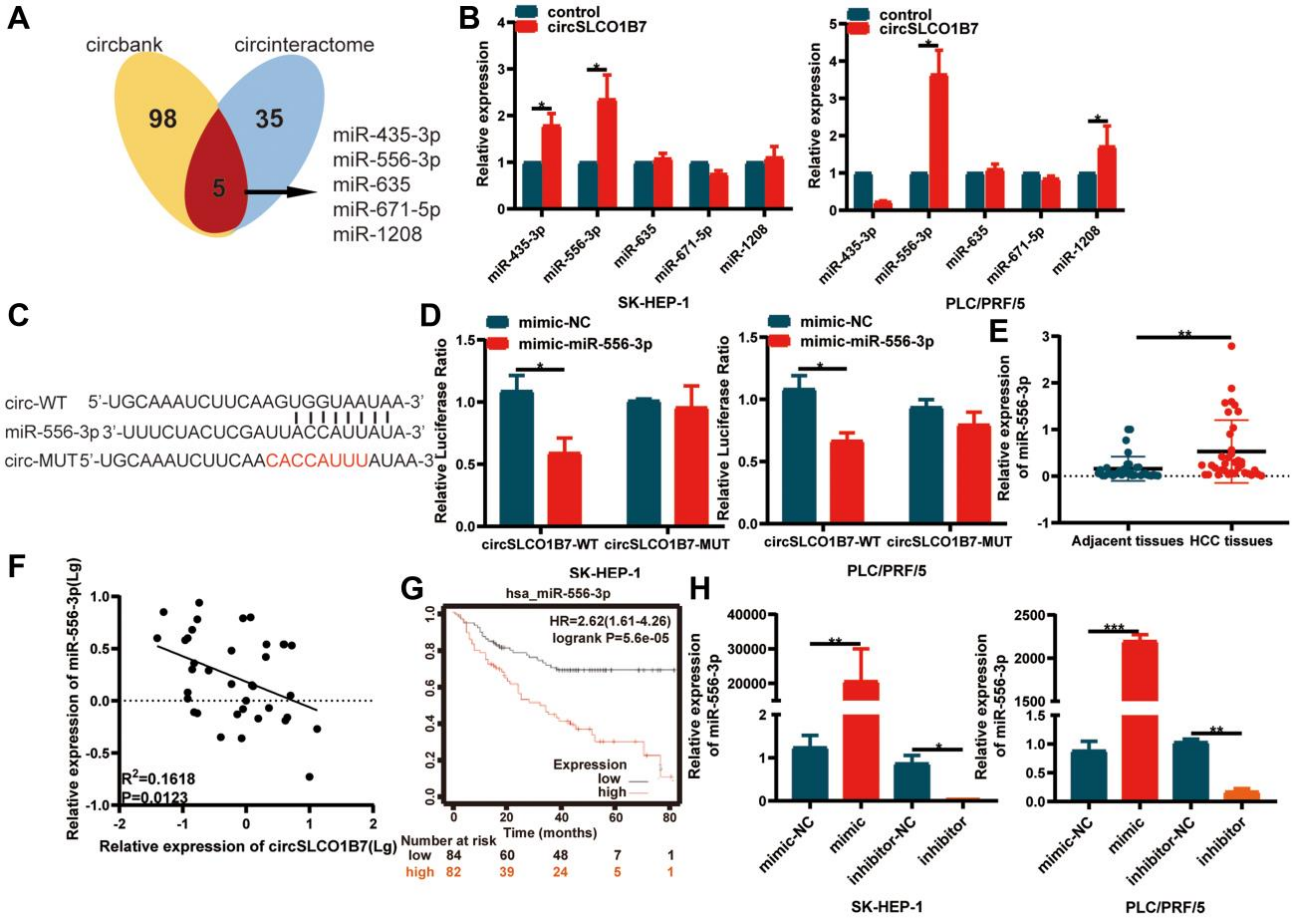
**circSLCO1B7 functions as miR-556-3p sponge.** (**A**) Bioinformatics analysis predicted the miRNAs that circSLCO1B7 might competitively bind to miRNAs. (**B**) RIP experiments were performed to identify the miRNAs to which circSLCO1B7 might competitively bind. (**C**, **D**) The luciferase activity of circ_0025583-WT or circ_0025583-MUT in the miR-556-3p mimic or NC in SK-HEP-1 and PLC/PRF/5 cells was shown. (**E**) The expression of miR-556-3p in HCC tissues was detected by qRT–PCR. (**F**) The expression of miR-556-3p was negatively related to circSLCO1B7 in HCC tissues. (**G**) The survival analysis of miR-556-3p was downloaded from Kaplan–Meier plotter (*n* = 166). (**H**) The transfection efficiency of the miR-556-3p mimic and inhibitor was measured by qRT–PCR. All data are presented as the means ± SD. ^*^P < 0.05, ^**^P < 0.01.

As competitive endogenous RNAs, the mechanism of circRNAs in sponging miRNAs to regulate downstream gene expression is widely studied. We used the Circular RNA Interactome (https://circinteractome.nia.nih.gov/) and circBank (http://www.circbank.cn/) databases to predict miRNAs. Taking the intersection of the results of the two databases, it was found that miR-435-3p, miR-556-3p, miR-635, miR-671-5p and miR-1208 were likely to bind to circSLCO1B7 (Figure 4A). We designed a biotin-coupled circSLCO1B7 probe and control probe to perform the RIP assay. The qRT–PCR results showed that the biotin-coupled circSLCO1B7 probe could enrich miR-556-3p more than other miRNAs (Figure 4B). A dual-luciferase reporter assay further verified the association between circSLCO1B7 and miR-556-3p. The circSLCO1B7 luciferase reporter plasmids with wild-type (circSLCO1B7-WT) or mutant-type (circSLCO1B7-MUT) and miR-556-3p mimic or NC were co-transfected into SK-HEP-1 and PLC/PRF/5 cells. The results showed that the miR-556-3p mimic combined with circSLCO1B7-WT could significantly reduce luciferase activity, while the circSLCO1B7-MUT had no significant effect on luciferase activity (Figure 4C, 4D).

Furthermore, we confirmed that the expression of miR-556-3p was higher in HCC tissues than in adjacent normal tissues (Figure 4E). In addition, we analyzed the correlation between circSLCO1B7 and miR-556-3p expression levels in HCC tissues. The miR-556-3p expression level was negatively correlated with circSLCO1B7 expression level (Figure 4F). To study the clinical value of miR-556-3p, we analyzed the data from Kaplan–Meier plotter (http://kmplot.com/analysis/). The median expression level of miR-556-3p in 166 HCC patients was used as the cut-off value, the survival rate of the low miR-556-3p group was obviously better than that of the high miR-556-3p group (Figure 4G). Thus, we designed miR-556-3p mimic and inhibitor and identified the transfection efficiency by qRT–PCR (Figure 4H). All these experiments showed that circSLCO1B7 could bind with miR-556-3p and act as a miR-556-3p sponge.

### circSLCO1B7 sponges miR-556-3p by targeting DAB2IP

**Figure 5:**
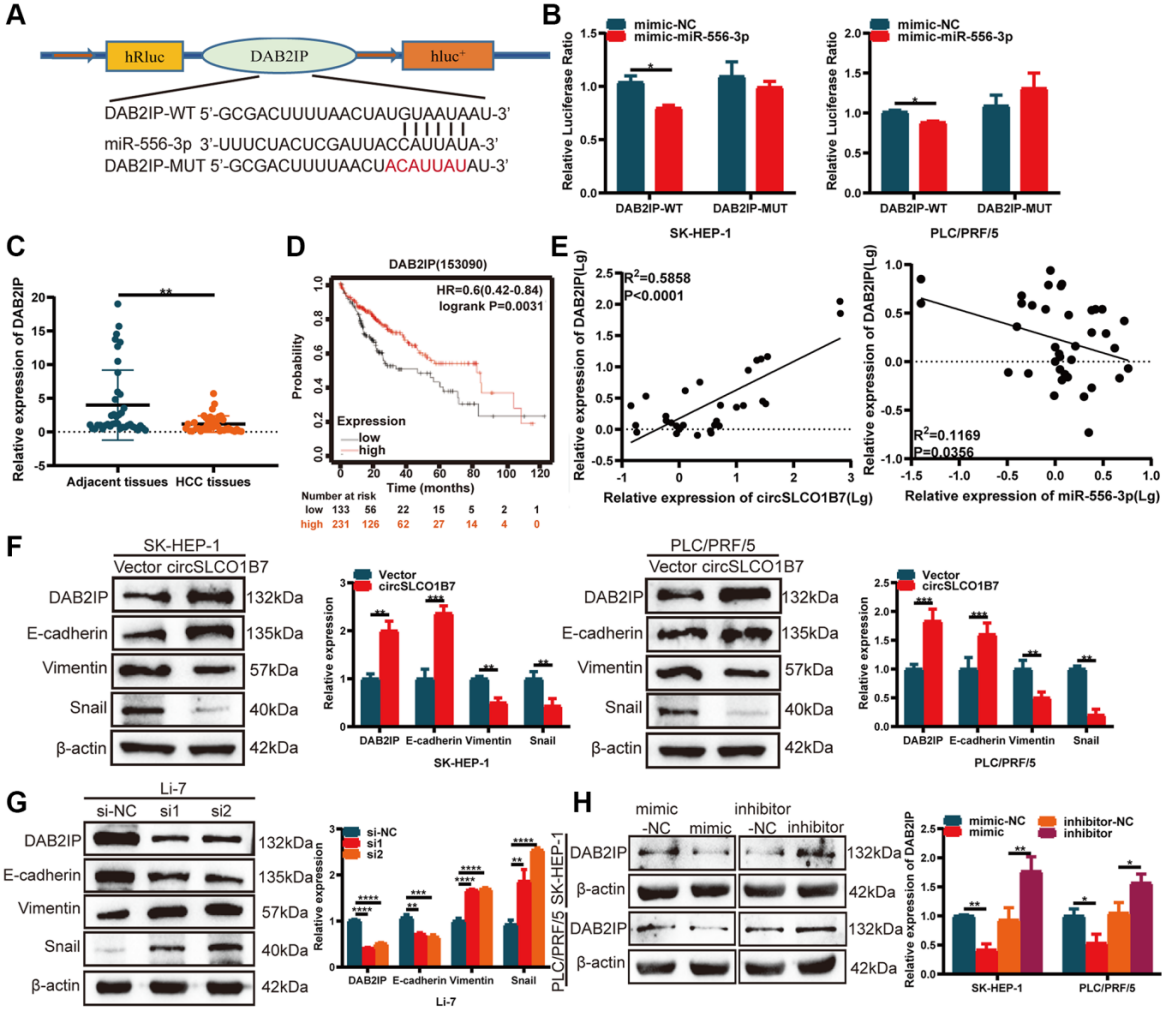
**circSLCO1B7 sponges miR-556-3p by targeting DAB2IP.** (**A**) The binding sites between DAB2IP and miR-556-3p. (**B**) The luciferase activity of SK-HEP-1 and PLC/PRF/5 cells transfected with the WT or MUT sequence of DAB2IP and miR-556-3p mimic-NC or the mimic is shown. (**C**) qRT–PCR was used to detect the expression of DAB2IP in HCC tissues. (**D**) The survival analysis of DAB2IP was downloaded from Kaplan–Meier plotter (*n* = 364). (**E**) The expression of DAB2IP was positively related to circSLCO1B7 in HCC tissues and negatively related to miR-556-3p. (**F**, **G**) Western blot analysis of the protein levels of DAB2IP, E-cadherin, Vimentin and Snail in SK-HEP-1 and PLC/PRF/5 cells when circSLCO1B7 was overexpressed or knockdown. (**H**) Western blot analysis of the protein levels of DAB2IP in SK-HEP-1 and PLC/PRF/5 cells when miR-556-3p was overexpressed or knockdown. All data are presented as the means ± SD. ^*^P < 0.05, ^**^P < 0.01, ^***^P < 0.001, ^****^P < 0.0001.

TargetScan (http://www.targetscan.org/), miRDB (http://www.mirdb.org/) and starBase (http://starbase.sysu.edu.cn/) were used to analyze the potential gene of miR-556-3p. After the intersection of these three databases, we finally selected DAB2IP as the target gene of miR-556-3p. A dual-luciferase reporter assay further verified the association between miR-556-3p and DAB2IP. The luciferase reporter plasmids with DAB2IP-WT or DAB2IP-MUT contained the binding sites of miR-556-3p (Figure 5A). The results showed that the miR-556-3p mimic combined with DAB2IP-WT groups could significantly reduce luciferase activity, while the DAB2IP-MUT had no significant effect on luciferase activity (Figure 5B). Then, we studied the expression of DAB2IP in HCC tissues, and the results showed that the expression of DAB2IP in HCC tissues was decreased (Figure 5C). Similarly, the survival curve of DAB2IP was downloaded from Kaplan–Meier plotter, and the follow-up results of 364 patients showed that patients with low DAB2IP expression had a longer survival time (Figure 5D). It was positively correlated with the expression of circSLCO1B7 and negatively correlated with the expression of miR-556-3p (Figure 5E). Finally, the circSLCO1B7-miR-556-3p-DAB2IP axis was constructed.

### circSLCO1B7-miR-556-3p-DAB2IP axis affects tumor metastasis

EMT is a hallmark of migration that allows polarized epithelial cells to acquire a stem-cell like mesenchymal phenotype, which contributes to cancer metastasis. Western blot analysis was implemented to compare the expression of EMT-related proteins. When circSLCO1B7 was overexpressed, the expression levels of DAB2IP and epithelial biomarker E-cadherin proteins were also significantly increased, while the expression levels of mesenchymal biomarkers Vimentin and Snail were decreased (Figure 5F). With the knockdown of circSLCO1B7, the expression level of E-cadherin protein was reduced, while the expression levels of Vimentin and Snail were increased (Figure 5G). To further evaluate the effect of miR-556-3p on DAB2IP expression, miR-556-3p mimic or inhibitor was transfected into SK-HEP-1 and PLC/PRF/5 cells. The results of Western blot showed miR-556-3p mimic decreased the expression level of DAB2IP while miR-556-3p inhibitor increased the expression level of DAB2IP (Figure 5H).

**Figure 6:**
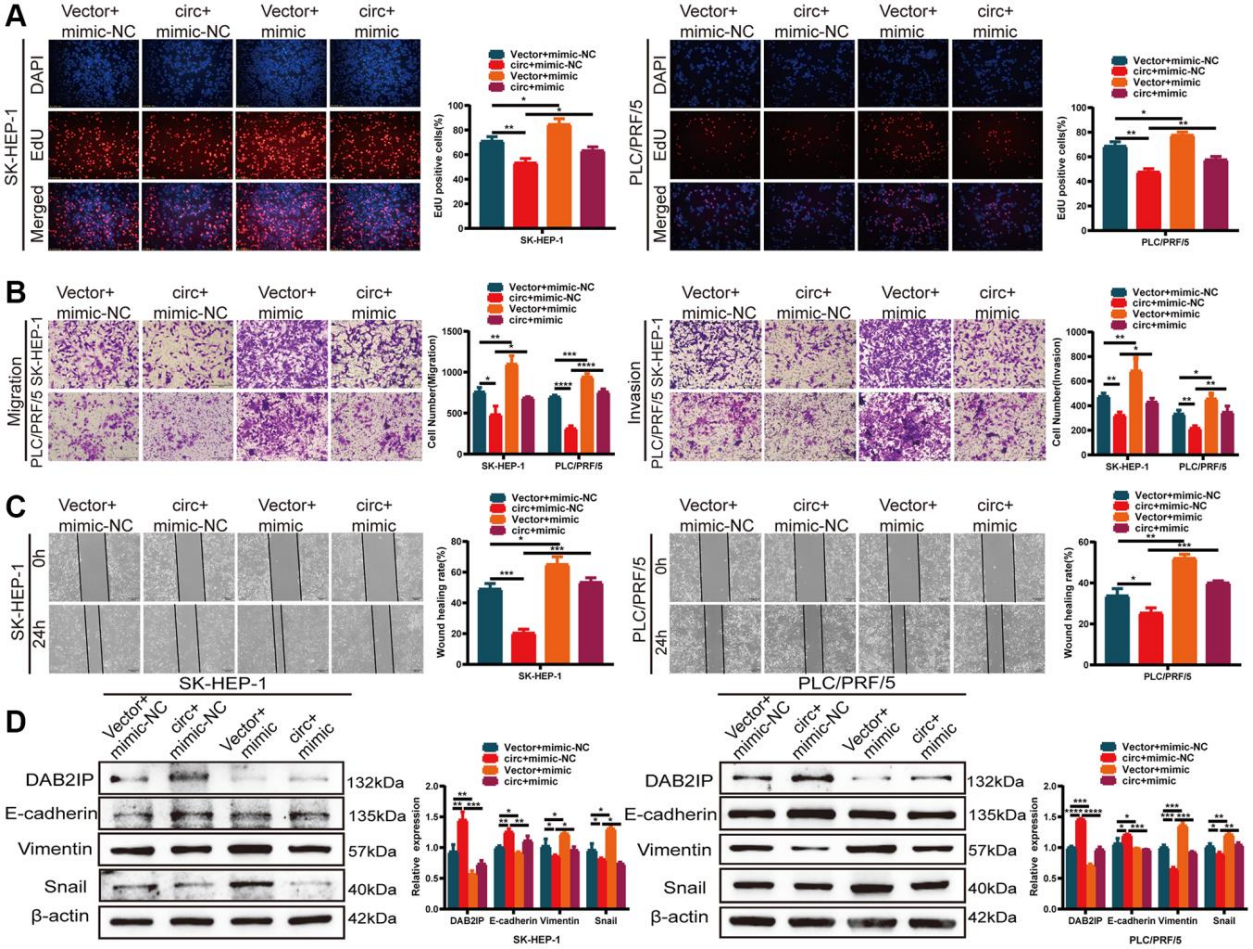
**circSLCO1B7 sponges miR-556-3p by targeting DAB2IP.** (**A**–**C**) The EdU assay (**A**), Transwell assay (**B**) and wounding healing assay (**C**) was used to detect the ability of proliferation, invasion and migration of HCC cells transfected with vector+mimic-NC, circ+mimic-NC, vector+mimic and circ+mimic. (**D**) Western blot analysis of the protein levels of DAB2IP, E-cadherin, Vimentin and Snail in SK-HEP-1 and PLC/PRF/5 cells transfected with vector+mimic-NC, circ+mimic-NC, vector+mimic and circ+mimic. All data are presented as the means ± SD. ^*^P < 0.05, ^**^P < 0.01, ^***^P < 0.001, ^****^P < 0.0001.

What’s more, we co-transfected HCC cells with circSLCO1B7-overexpressing plasmids and the miR-556-3p mimic. We constructed four groups: Vector+mimic-NC, circ+mimic-NC, Vector+mimic and circ+mimic. EdU assays, Transwell assays and wound healing assays were performed. The results showed that the proliferation, invasion and migration abilities of HCC cells were decreased in circ+mimic-NC group and increased in the Vector+mimic group (Figure 6A–6C). The miR-556-3p mimic could antagonize the effect of circSLCO1B7 in inhibiting tumor metastasis. Western blot analysis showed that overexpression of circSLCO1B7 increased the expression levels of DAB2IP and E-cadherin proteins and reduced the expressions levels of Vimentin and Snail proteins. Moreover, miR-556-3p mimic could reverse this effect (Figure 6D). Thus, these data indicated that circSLCO1B7 could affect tumor metastasis by sponging miR-556-3p to regulate the expression of DAB2IP.

### Overexpression of circSLCO1B7 inhibits the growth of HCC cells *in vivo*

**Figure 7:**
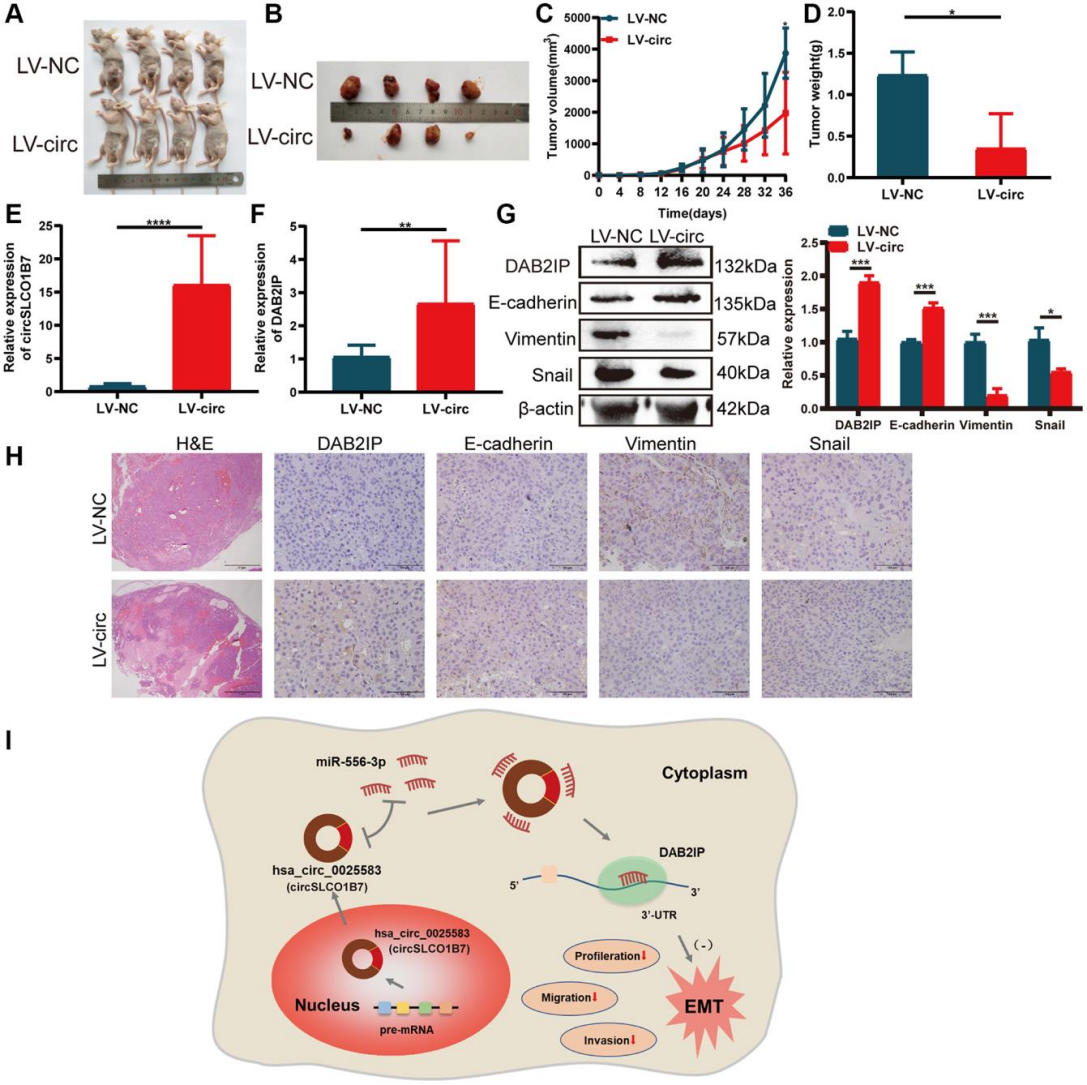
**Overexpression of circSLCO1B7 inhibited the growth of HCC cells *in vivo*.** (**A**, **B**) Nude mice were injected with LV-control and LV-circ (circSLCO1B7) PLC/PRF/5 cells. After 36 days, the nude mice and tumor tissues were treated and photographed. (**C**) The size of tumor tissues was measured every 4 days. The growth curve was made after 36 days. (**D**) The weight of tumor tissues was measured. (**E**, **F**) qRT–PCR was used to detect the expression of circSLCO1B7 and DAB2IP in the LV-circ group compared to the LV-control group. (**G**) Western blot analysis of the protein levels of E-cadherin, Vimentin and Snail in the LV-circ group compared to the LV-control group. (**H**) HE and IHC experiments showed the difference between the LV-circ group and LV-control group and the expression of DAB2IP, E-cadherin, Vimentin and Snail. (**I**) A schematic diagram on the mechanism of circSLCO1B7 affecting the progression of HCC. All data are presented as the means ± SD. ^*^P < 0.05, ^***^P < 0.001, ^****^P < 0.0001.

To study the effect of circSLCO1B7 on the growth of HCC, we established the model of subcutaneous tumorigenesis. Stably transfected lentiviral vectors (LV-NC and LV-circ) were injected into the armpit of mice. A total of 8 mice were divided to LV-NC group and LV-circ group. 36 days later, the tumors in the LV-circ group were significantly smaller than those in the LV-NC group (Figure 7A, 7B). Overexpression of circSLCO1B7 reduced both the volume and weight of the tumors (Figure 7C, 7D). The expression levels of circSLCO1B7 and DAB2IP were increased in the LV-circ group (Figure 7E, 7F). H&E staining revealed that there were few tumor foci of HCC in the LV-circ group compared to the LV-NC group. IHC and Western blot results showed that the expression levels of Vimentin and Snail were reduced and the expression levels of DAB2IP and E-cadherin were enhanced in the LV-circ group (Figure 7G, 7H). Thus, we concluded that overexpression of circSLCO1B7 inhibited the growth of HCC *in vivo*.

## DISCUSSION

According to the differential expressions in different cancers, circRNAs can be divided into oncogenes and anti-oncogenes, which promote or inhibit tumor growth and metastasis, respectively. In our study, the expression of different circRNAs in 5 pairs of HCC tissues was analyzed by high-throughput sequencing, and circSLCO1B7, with the most obvious expression differences, was selected for further study. qRT–PCR verified that circSLCO1B7 showed low expression in both 50 pairs of HCC tissues and five kinds of HCC cells. circSLCO1B7 expression was correlated with the TNM stage, lymph node metastasis and tumor size of HCC patients, but not with patient age, sex or serum AFP. These results showed that circSLCO1B7 may be helpful to assess the prognosis of HCC. Our results also found that the expression of circSLCO1B7 in plasma of 50 HCC patients was lower compared to normal healthy people. Previous studies have found that circRNAs can be stably expressed in plasma. Researchers reported that hsa_circ_0001445 was highly expressed in plasma of 104 HCC patients, and it was more accurate than AFP in diagnosing HCC, with an AUC of 0.862 [21]. The clinical sample size of this study was small, and the diagnostic accuracy of circSLCO1B7 still needs to be further studied.

We have demonstrated that circSLCO1B7 had a circular structure and was not degraded by RNase-R enzyme. These properties made circSLCO1B7 valuable for further function study. The effects of circSLCO1B7 overexpression and knockdown on apoptosis, cell cycle, proliferation, migration and invasion were studied *in vivo* and *in vitro*, respectively, indicating that circSLCO1B7 can be used as a tumor suppressor gene to inhibit tumor metastasis.

The most remarkable function of circRNAs was to be used as a sponge for miRNA to regulate the expression of downstream target genes. For example, Guo et al. demonstrated that circ3823, which acts as an oncogene, promotes the growth and metastasis of colorectal cancer. Mechanistically, circ3823 competitively combined with miR-30c-5p to regulate TCF7 expression [22]. Wang et al. found that circWAC was upregulated in breast cancer to activate the circWAC/miR-142/WWP1 axis. The axis obviously induced chemotherapeutic resistance and thus predicted poor prognosis [23]. Then, we used bioinformatics and RIP experiments to identify that miR-556-3p was the most closely associated with circSLCO1B7, and a dual-luciferase reporter assay confirmed the correlation between circSLCO1B7 and miR-556-3p expressions. According to previous studies, miR-556-3p is highly expressed in haemangioma cancer and HCC [24, 25]. MiR-556-3p was closely associated with tumor metastasis. qRT–PCR results revealed that miR-556-3p was highly expressed in HCC tissues compared with adjacent tissues and was negatively correlated with the expression of circSLCO1B7. Rescue experiments showed that overexpression of miR-556-3p could reverse the inhibitory effect of circSLCO1B7 on the migration and invasion of HCC cells. Then, we used three databases (miRDB, TargetScan and starBase) to screen out the target gene DAB2IP, which had low expression in HCC tissues. It was reported that DAB2IP was downregulated in HCC and played an important role in EMT process, tumor migration and invasion [26, 27]. In summary, circSLCO1B7/miR-556-3p/DAB2IP axis was conducted.

Clinically, patients are often complicated with intrahepatic metastasis and distant metastasis, resulting in increased mortality of HCC patients. Therefore, active exploration of HCC metastasis targets is the focus of research. Our results suggested that circSLCO1B7 inhibited the invasion and migration ability of HCC cells and was closely correlated with TNM stage, lymph node metastasis and tumor size. What’s more, DAB2IP was involved in the EMT process. The EMT mechanism causes epithelial cells to gradually lose their identity and transform into mesenchymal cells. The EMT process mainly marked the downregulation of E-cadherin and upregulation of Vimentin and Snail. Thus, we further studied the role of the circSLCO1B7/miR-556-3p/DAB2IP axis in EMT process. Both overexpression of circSLCO1B7 and knockdown of miR-556-3p increased the protein level of DAB2IP and delayed the EMT process, while knockdown of circSLCO1B7 and overexpression of miR-556-3p had the opposite effects. CircSLCO1B7/miR-556-3p/DAB2IP axis inhibited the malignant progression of HCC. CircRNAs could bind proteins directly to regulate cellular metabolism, regulate immunity and be modified by N6-methyladenosine [10, 28, 29]. The other mechanism of circSLCO1B7 in the development of HCC may be further explored.

In summary, circSLCO1B7 is lowly expressed in HCC tumor tissues and cells. Overexpression of circSLCO1B7 significantly can inhibit HCC cell proliferation, arrest the cell cycle and decrease cell invasion and migration. CircSLCO1B7 can affect the malignant progression of HCC as a sponge of miR-556-3p to regulate the target gene DAB2IP. The circSLCO1B7/miR-556-3p/DAB2IP axis is constructed to influence the malignant progression of HCC (Figure 7I).

## MATERIALS AND METHODS

### Clinical samples

The subjects including tissues and plasma of 50 HCC patients and 50 healthy people were collected from Nantong Third Hospital Affiliated to Nantong University from 2019 to 2020. These tissues and plasma were stored in the refrigerator at −80°C immediately after being removed from the subjects. The average age of HCC patients was 60 years (40–80 years). The studies involving human participants were reviewed and approved by the Ethics Committee of Nantong Third Hospital Affiliated of Nantong University. The patients provided their written informed consent to participate in this study.

### Quantitative reverse transcription-polymerase chain reaction (qRT–PCR)

TRIzol^®^ reagent (Invitrogen; Thermo Fisher Scientific, Inc., USA) was used to extract total RNA from tumor tissues, cell lines and plasma according to the manufacturer’s instructions. cDNA was formed by reverse transcription using PrimeScript RT Master Mix (Takara Bio, Japan). SYBR Green Master Mix (Vazyme Biotech Co., Ltd., China) and a Bio-Rad Real-Time qRT–PCR detection system (Bio-Rad Laboratories, Inc., USA) were used to detect gene expression for qRT–PCR. The sequences were shown in Supplementary Table 1. All experiments were repeated three times.

### Cell culture and transfection

Normal hepatocyte L-02 and HCC cell lines such as SK-HEP-1, PLC/PRF/5, Li-7, Huh-7 and Hep3B2.1-7 were purchased from the Chinese Academy of Sciences (Shanghai, China). The cell medium was MEM medium (Gibco; Thermo Fisher Scientific Inc.) containing 10% FBS for 5% CO_2_ at 37°C. The circSLCO1B7 overexpression plasmid, small interfering RNA (siRNA) and normal control (NC) were obtained from Suzhou GenePharma Co., Ltd., (China) and the hsa_miR-556-3p mimic and inhibitor were obtained from RiboBio Co., Ltd. (China). Cells were transfected with Lipofectamine^®^ 3000 (Invitrogen; Thermo Fisher Scientific, Inc.,) according to manufacturer’s instructions. The sequences of siRNAs were shown in Supplementary Table 1. All experiments were repeated three times.

### Nuclear and cytoplasmic extraction and fluorescence *in situ* hybridization (FISH)

The content of RNAs in cytoplasm and cytoplasm was determined using an RNA Subcellular Isolation kit (Invitrogen, Thermo Fisher Scientific Inc., USA). FISH was used to determine the location of circSLCO1B7 and miR-556-3p in PLC/PRF/5 cells. A total of 100 μl of circSLCO1B7 probe, miR-556-3p probe and 18 S probe mixture (4 μM) were prepared to cover the surface of the slipper overnight at 37°C. The slipper was stained with DAPI and immediately observed under a fluorescence microscope (Olympus Corporation, Japan). The sequences were shown in Supplementary Table 2. All experiments were repeated three times.

### RNase R assay

RNase R (3 U/μg) was incubated with total RNA at 37°C for 5 min and 70°C for 10 min. qRT–PCR was used to compare the expression of circSLCO1B7 between the RNase R-treated group and the non-treated group. All experiments were repeated three times.

### Flow cytometry assay

At 48 h after transfection, the cells for apoptosis assay were incubated with 5 μl of Annexin V and 5 μl of 7-AAD. The cells for cell cycle assay were stained with 300 μl of PI with 20 μg/ml RNase A (Sigma-Aldrich; Merck KGaA, Germany). The apoptosis rate and cell cycle distribution were measured by a FACSCalibur flow cytometer (BD Biosciences, USA) and counted by FlowJo software 10.7 (FlowJo LLC, USA). All experiments were repeated three times.

### Cell proliferation assay

Cell Counting Kit-8 assay (CCK-8; Dojindo Laboratories, Japan) and 5-Ethynyl-20-deoxyuridine (EdU; RiboBio, China) incorporation assay were used to detect the ability of cell proliferation. For CCK-8 assay, 2 × 10^3^ cells were seeded into 96-well plates. After culturing for 0 h, 24 h, 48 h and 72 h, the cells were incubated with 10 μl CCK-8 for 4 h. The absorbance at 450 nm was measured by a spectrophotometer. For EdU assay, the stained cells were photographed with an Olympus IX73-FL-PH fluorescence microscope (Olympus, Japan). All experiments were repeated three times.

### Migration and invasion assay

Transwell assay and wounding healing assay could effectively detect the ability of cells to invade and migrate. For Transwell assay, a total of 2 × 10^5^ transfected cells in serum-free medium were seeded into the upper chamber (pore size, 3 μm; Millipore Sigma, USA). The lower chamber was filled with complete medium with 20% FBS. The quantity of cells that were stained with 1% crystal violet for 10 min could be counted using an Olympus IX73-FL-PH inverted microscope (Olympus, Japan). For wounding healing assay, a straight line was drawn on the plate with a 10-μl pipette tip. Cell growth was photographed separately in the same location at 0 h and 24 h respectively using an Olympus IX73-FL-PH inverted microscope (Olympus, Japan). All experiments were repeated three times.

### RNA immunoprecipitation (RIP)

Cells were transfected with biotin-couple circSLCO1B7 probes and control probes for 24 h at 37°C. After lysis, ultrasonic crushing, and centrifugation, the cell suspension was reversed and mixed with magnetic beads (Life Technologies, USA) overnight at 30°C. RNAs binding to magnetic beads were extracted using TRIzol^®^ reagent for qRT–PCR. All experiments were repeated three times.

### Dual luciferase reporter assay

Wild-type or mutant circSLCO1B7 and DAB2IP were designed according to their sequences, and miR-556-3p mimic-NC or mimic were co-transfected into the cells. The luciferase activity was determined by a dual-luciferase reporter assay system (Promega, USA) according to the manufacturer’s protocol and detected using a Lumipro system (Atila BioSystems, Inc., USA). All experiments were repeated three times.

### Immunohistochemical staining (IHC)

The 4-μm tissue slices were made and incubated with anti-DAB2IP (1:200; cat. no. ab87811; Abcam, UK), anti-E-cadherin (1:400; cat. no. 3195S; Cell Signaling Technology, Inc., USA), anti-Vimentin (1:100; cat. no. 5741S; Cell Signaling Technology, Inc.) and anti-Snail (1:1000; cat. no. NBP2-27293; Novus Biologicals, Inc., USA) overnight, and then the mixture was incubated with HRP-conjugated secondary antibody for 30 min at room temperature, dyed by DAB and observed under a fluorescence microscope. All experiments were repeated three times.

### Western blot

The proteins in the samples were extracted using RIPA lysis buffer (Beyotime Institute of Biotechnology, China) containing PMSF. After electrophoresis, the proteins were transferred to a polyvinylidene difuoride (PVDF) membrane. After blocking in non-fat milk, the membrane was incubated with primary antibodies overnight at 4°C and washed thoroughly three times with TBST-Tween-20 (0.1%). After incubation with HRP-conjugated secondary antibody for 1 h at room temperature, the bands were observed by Tanon. All experiments were repeated three times.

### Animal experiments

BALB/c nude mice (4 weeks old, *n* = 8) were purchased from The Animal Care Committee of Nantong University and reared under standard conditions. These mice were randomly divided into LV-NC group and LV-circ group. PLC/PRF/5 cells (1 × 10^7^) with LV-NC and LV-circ were resuspended in PBS and subcutaneously injected in the right thigh of nude mice. The formula was: volume = 0.5 × length × width^2^. 30 days later, the tumor tissues were harvested for qRT–PCR, Western blot and IHC. The animal study was reviewed and approved by the Animal Care Committee of Nantong University. The experiment was conducted without blind experimentation. All experiments were repeated three times.

### Statistical analysis

All results were shown as the mean ± SD, and all the data analyses were carried out using GraphPad Prism 8.0 (GraphPad Software Inc., USA). Student’s *t* test was used to compare the difference between the two groups. One-way ANOVA and Tukey’s test were used for multiple comparisons. *P* < 0.05 was considered statistically significant. None of samples or animals were excluded from the analysis. The investigator was not blinded to the group allocation during the experiments. There is no estimate of variation in each group of data. The variance is similar between the groups. The data were excepted to meet the normal distribution.

## Supplementary Materials

Supplementary Tables
