# Supplementary material for: circSLCO1B7 suppresses the malignant progression of hepatocellular carcinoma via the miR-556-3p/DAB2IP axis
Source: Aging (Albany NY). 2023 Nov 24;15(22):13329–44. doi: 10.18632/aging.205244 (PMC10713434; doi:10.18632/aging.205244)
Supplement: Supplementary Tables [file aging-15-205244-s001.pdf]

SUPPLEMENTARY TABLES

Supplementary Table 1. Sequences of the qRT-PCR primers and siRNA used in this study.

Gene name	Forward primer (5′–3′)	Reverse primer (5′–3′)
circSLCO1B7	ACGTGTATGAAGTGGTCCACCA	ACCAAGCCACCAAGCTCCAA
SLCO1B7	CCGGCCTAACCTTGACCTATGATG	ACAGACGGGTTCCCATTGACTTTC
DAB2IP	GCGGCAGTTCGTGGAGAAGTG	GGCAGGATGGTGATGGTTTGGTAG
18srRNA	GTAACCCGTTGAACCCCATT	CCATCCAATCGGTAGTAGCG
β-actin	GAGAAATCTGGCACCACACC	GGATAGCACAGCCTGGATAGCAA
GAPDH	GGTATGACAACGAATTTGGC	GAGCACAGGGTACTTTATTG
si-NC	UUCUCCGAACGUGUCACGUTT	ACGUGACACGUUCGGAGAATT
circSLCO1B7 si1	CCACAUAUUUGGGGCACUATT	UAGUGCCCCAAAUAUGUGGTT
circSLCO1B7 si2	CAUAUUUGGGGCACUAUCATT	UGAUAGUGCCCCAAAUAUGTT

Supplementary Table 2. Sequences of the FISH probes used in this study.

Gene name	Probe (5′–3′)
circSLCO1B7	Cy3-GTTATCCTGATAGTGCCCCAAATATGTGGA
18srRNA	Cy3-CTTCCTTGGATGTGGTAGCCGTTTC
